# A groundwork for allostatic neuro-education

**DOI:** 10.3389/fpsyg.2015.01224

**Published:** 2015-08-17

**Authors:** Lee Gerdes, Charles H. Tegeler, Sung W. Lee

**Affiliations:** ^1^Brain State Technologies LLCScottsdale, AZ, USA; ^2^Department of Neurology, Wake Forest School of MedicineWinston-Salem, NC, USA; ^3^Running River SchoolSedona, AZ, USA

**Keywords:** allostasis, neuro-education, neurodevelopment, RDoC, executive function, polyvagal theory, toxic stress, neurotechnology

## Abstract

We propose to enliven educational practice by marrying a conception of education as guided human development, to an advanced scientific understanding of the brain known as allostasis (stability through change). The result is a groundwork for allostatic neuro-education (GANE). Education as development encompasses practices including the organic (homeschooling and related traditions), cognitive acquisition (emphasis on standards and testing), and the constructivist (aimed to support adaptive creativity for both learner and society). Allostasis views change to be the norm in biology, defines success in contexts of complex natural environments rather than controlled settings, and identifies the brain as the organ of central command. Allostatic neuro-education contrasts with education focused dominantly on testing, or neuroscience based on homeostasis (stability through constancy). The GANE perspective is to view learners in terms of their neurodevelopmental trajectories; its objective is to support authentic freedom, mediated by competent, integrated, and expansive executive functionality (concordant with the philosophy of freedom of Rudolf Steiner); and its strategy is to be attuned to rhythms in various forms (including those of autonomic arousal described in polyvagal theory) so as to enable experiential excitement for learning. The GANE presents a variety of testable hypotheses, and studies that explore prevention or mitigation of the effects of early life adversity or toxic stress on learning and development may be of particular importance. Case studies are presented illustrating use of allostatic neurotechnology by an adolescent male carrying diagnoses of Asperger’s syndrome and attention-deficit hyperactivity disorder, and a grade school girl with reading difficulties. The GANE is intended as a re-visioning of education that may serve both learners and society to be better prepared for the accelerating changes of the 21st century.

## Introduction and Orientation

One should not ask, ‘What does a person need to know and be able to do for the existing social order?’ but rather, ‘What gifts does a person possess and how may these be developed?’ Then it will be possible to bring to society new forces from each succeeding generation. Then the social order will always be alive with that which fully developed individuals bring with them into life, rather than that each succeeding generation be made to conform to an existing social organization – Rudolf Steiner, Founder of Waldorf Schools.

Fenner and Rapisardo (1999)

Today, clinicians are accused of overdiagnosing and overmedicating children with behavioral problems. It is time to shift from an exclusive focus on behavior and symptom-based diagnosis to incorporate a deeper understanding of neurodevelopmental trajectories with interventions that can support the healthy development of brain and behavior – Thomas Insel, Director of the US National Institute of Mental Health.

Insel (2014)

**FIGURE 1:**
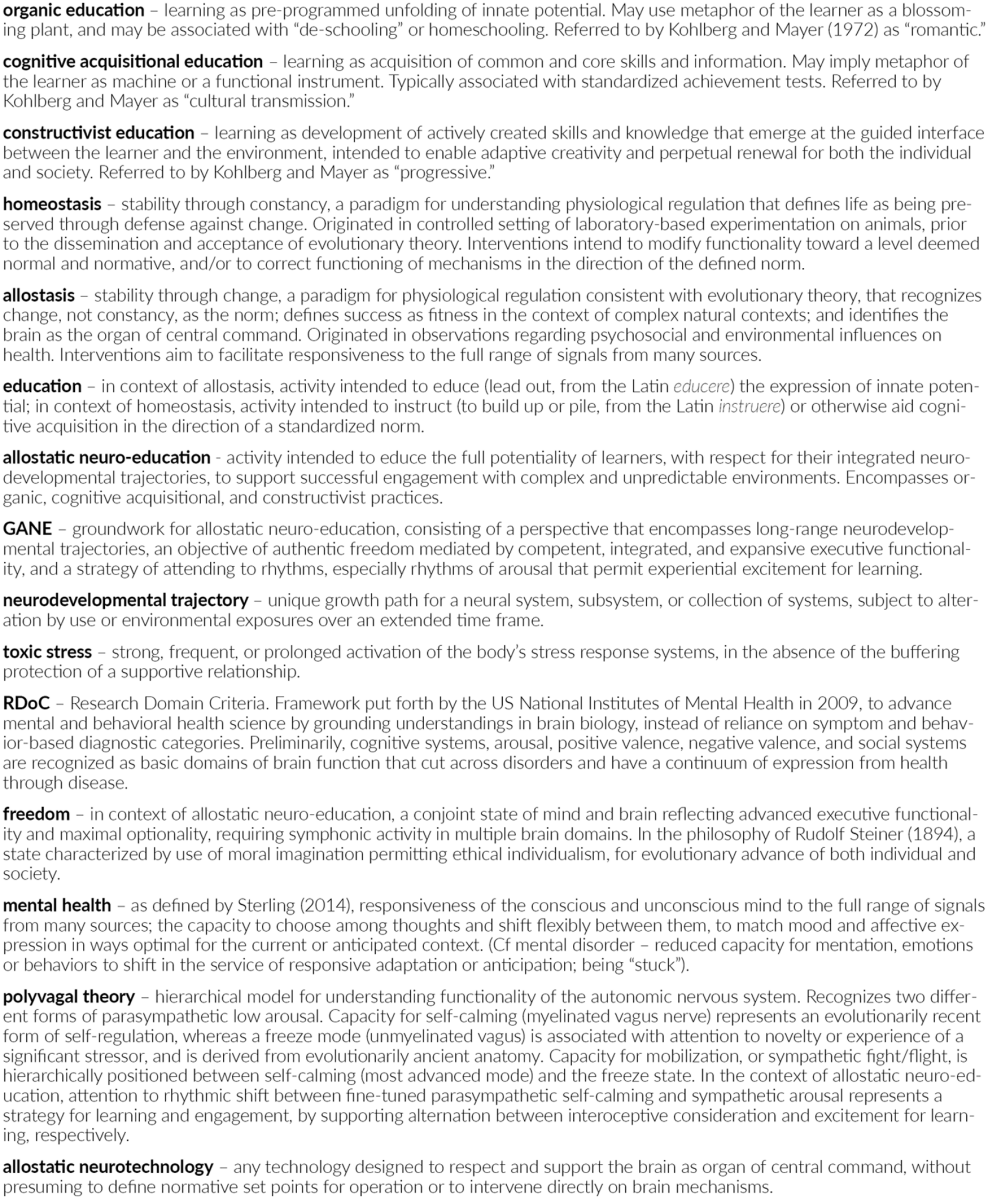
**Glossary of terms, concepts, and abbreviations presented in the paper, listed in the order in which they are principally discussed in the text**.

Educational practice is as old as human civilization. Modern understanding of the brain, supported by innovations in technology, has developed over approximately the last 100 years. The proposition of this paper is that a recent innovation in scientific understanding of the brain known as *allostasis* (“stability through change”) has potential to support a constructive re-visioning of education as stewardship of emergent, individualized, and advanced brain functionality. The progeny of the union between education and allostasis is an approach to the developing and multi-faceted learner that we call allostatic neuro-education. To characterize this conceptualization we outline a perspective, an objective, and a strategy for educators which collectively constitute a *groundwork for allostatic neuro-education* (GANE). The ultimate benefit we conceive from the GANE is to support the profession of education to reach its own highest potential, to be a match for the meaning reflected in its Latin etymology *educere*, to *lead out* the expression of that which is within. To support understanding of the terms, concepts, and abbreviations presented in this paper, a glossary is provided in **Figure 1**.

For purposes of introducing the reconstructed view of education entailed by the GANE, we recapitulate a taxonomy outlined by Lawrence Kohlberg, the pioneer of moral psychology, and Rochelle Mayer in a paper titled “Development as the Aim of Education” (Kohlberg and Mayer, 1972). In this work, Kohlberg and Mayer categorized educational practice in the Western world to consist of three broadly different streams or typologies – the romantic, the transmission of culture, and the progressive. To update their labels, we substitute the words *organic* for their *romantic*, *cognitive acquisition* for their *cultural transmission*, and *constructivist* for their *progressive*.

The *organic* type of educational practice is intended to nurture that which is already within the child. Cognitive development is encouraged (rather than instructed) to proceed along lines of pre-programmed unfolding and in parallel with physical and emotional development. Organic education is philosophically descended from Jean Jacques Rousseau (1712–1778) and others who exalt the natural and express concern over the corrupting influences of society. Formal and didactic aspects of education tend to be de-emphasized in favor of child-centric practices including homeschooling, de-schooling, and other strategies aimed to enable flourishing of innate potential. Education for *cognitive acquisition* is rooted in classical traditions of Western civilization, aiming to impart literacy, mathematical competence, and other forms of knowledge that relate to prevalent norms. Education for cognitive acquisition is largely society-centric, and it is largely synonymous with education as instruction (from the Latin *instruere*, to build up or pile on) that includes emphases on standardized testing. In the USA, coalescence around standards of cognitive skill acquisition has occurred in various ways including federal legislation known as No Child Left Behind as well as the more recent initiative known as the Common Core. In East Asian cultures, cognitive acquisition takes on even greater importance in that educational striving is commonly used as a tool for character building or as a high-stakes funnel for social advancement (Chua, 2011; Ripley, 2014). *Constructivist* education, represented by John Dewey (1859–1952) and others, posits that the goal of education is to facilitate guided development of the learner through proactive experiences and interactions with society and the natural environment at large, such that knowledge and skills are actively created through engagement with living and changing contexts. Dewey in particular considered that education to nurture such development should have implications for both the learner and for healthy participatory democracy.

Kohlberg and Mayer associated the first two educational streams with different metaphors for learning. Organic education compares the learner to a plant or blossoming flower. For education in the service of cognitive acquisition, the learner has inputs and outputs, comparable to a machine or other functional instrument. For the constructivist, the learner is understood to be engaged in a constant dialectic with the environment. She or he generates and applies an initial idea, experiences its consequences, and subsequently revises the idea or its application in a continuous procession. Kohlberg and Mayer were proponents of constructivism, proposing that this approach was the only one adequate to “prepare free people for factual and moral choices which they will inevitably confront in society.” Their sentiment is echoed in other recent writing that has warned against the societal impacts of educational systems that begin and terminate with unbridled focus on cognitive acquisition. Such approaches can fail to recognize fundamental misalignments between learner needs and teacher efforts, ultimately resulting in wasted resources and distorted understanding, akin to “lecturing birds how to fly” (Taleb, 2012).

The GANE in contrast recognizes important roles for all three educational streams or traditions, and the dialectical appreciation that Kohlberg and Mayer assigned exclusively to the constructivist can be understood to apply to all three traditions aligned in a larger unfolding. The child-centric view of organic education can be understood, normatively, as a robust starting point for the learner, as a way to nurture the innate capacities of a young life form. The society-centric view of education for cognitive acquisition may serve to equip and ballast developing learners with a common set of shared skills and knowledge which enable clear and robust communal interaction, without which self-centered individuals may descend into narcissism or chaos. Though constructivism may represent the highest educational ideal, in its goal of enabling perpetual renewal and re-creation for both individual and society, actual constructivist practice may need a foundation of nurturing, experience, skills, and knowledge that are the province of organic and cognitive acquisitional approaches. The GANE thus aims for a re-visioning of education that is inclusive of a breadth of traditions.

The GANE is motivated by a sense of urgency for educators to consider seriously the need for innovation in educational principles and practice. Because of the acceleration of changes that we are now experiencing in multiple realms – socio-economic, cultural, geo-political, and technological – educational systems must better prepare 21st century learners to be adaptable and genuinely constructivist, so they may thrive in a world that is manifesting increasing degrees of variability and unpredictability. To further explicate the background of the GANE and how it differs from other approaches to neuro-education, the ensuing section of the paper introduces the advanced model of physiological regulation known as *allostasis*. Allostasis is proposed as a necessary advance on the paradigm of homeostasis, that has limitations associated with its origins in pre-evolutionary 19th century biology. The third section defines and characterizes the GANE by outlining its perspective, objective, and strategy. Fourth, the paper considers a range of testable hypotheses that stem from the GANE. To emphasize that the brain-centrism of the GANE is not metaphorical or only implied, two case studies are presented that illustrate use of allostatic neurotechnology by learners with special needs.

## Homeostasis, Allostasis, and Education

The advanced understanding of brain functionality that is central to the GANE is contained in the paradigm of *allostasis* (Sterling and Eyer, 1988), defined as “stability through change.” In comparison to the idea of homeostasis, or “stability through constancy,” allostasis presents a more biologically accurate understanding of the role and functionality of the brain itself (Sterling, 2004, 2012). There are three main insights from the paradigm of allostasis that enable it to serve as a robust scientific foundation for neuro-education. First, it recognizes that change and not constancy is the fundamental character of biological regulation. Second, it views life and the definition of success against the background of complex natural contexts rather than controlled settings. Third, allostasis identifies the brain as the organ of central command.

The innovative view of allostasis may be better understood by an overview of the paradigm it aims to subsume. The foundations of modern experimental physiology were laid by Claude Bernard (1813–1878) and Walter Cannon (1871–1945) through the use of laboratory-based animal experimentation methods and reasoning based on reductive materialism. Bernard posited that physiological systems are designed to preserve a constant interior environment – that is to say, that the goal of life forms is to defend their internal biological operations against changes wrought by the environment. Central to the paradigm was the presumption that differences between species were not of great significance, and thus inferences about the functioning of physiological systems drawn from experiments on one species could be applied to others. Homeostasis recognizes the individual as a collection of multiple and distinct systems – the heart and circulatory system, the gut and digestive system, and others – and that illumination of the operative mechanisms of these systems is the basis for therapeutics.

In contrast, the paradigm of allostasis was born from recognition that social and environmental variables have critical influence on biological functioning, and that life is not fundamentally oriented toward “defense” of a constant internal environment (Sterling, 2004, 2012). Rather, in alignment with evolutionary theory, biological systems are understood to function in such way that they normatively change their operational set points to be optimally fit for the context of the natural environment, which is essentially complex, variable, and unpredictable. *Under evolutionary theory, the ultimate goal of life is not to defend against changes but rather to create more life*, and this objective is achieved through adaptation to present and anticipated needs. Critically, allostasis recognizes that to coordinate and adjudicate among the competing demands of different organ systems or functionalities, life requires a higher-order faculty for organization and regulation, and the organ for such faculty is the brain. The brain is the seat of central command, guiding the organism as a unit to adapt to new circumstances, and to prepare for changes yet to come.

Biological systems under conditions of environmental monotony (including the controlled settings of a laboratory) may appear to be homeostatic because their set points are not challenged to need modification. Nonetheless homeostasis and allostasis present different understandings of the underlying drivers of physiological regulation and their implications. Homeostasis implies the existence of normal and normative set points, whereas allostasis does not (Sterling, 2012, 2014). Under allostasis, recognition that brain activity is fundamentally context-dependent is critical to understanding health or disease across systems, whereas under homeostasis the focus is on identifying disturbances in molecular mechanisms. Homeostatic modeling invites strategies to modify mechanisms and their set points toward the putative norms. Allostatic modeling recognizes the need for variability in biological expression and specifically aims to support the brain’s native ingenuity for central command (Sterling, 2004, 2014). Notably it is not the anatomical focus *per se* of a particular strategy that makes it homeostatic or allostatic, and interventions directed toward the brain can be of either type.

Dominance of the homeostatic paradigm in physiology and biomedical health care can be considered the neuroscientific analog of the dominance of standardized cognitive acquisition as the mode of educational practice in the modern era. Just as the modern biomedical system views the individual as so many distinct organ systems, so does educational practice for cognitive acquisition view the learner in terms of a collection of different and distinct systems for cognitive, emotional, and behavioral functionalities, and others. As with homeostatic reductionism, these systems are held to have independent mechanisms of operation, and in general only the cognitive is under the purview of the secular educator. Other systems are allocated to an array of specialists– psychologists, psychiatrists, other behavioral specialists, physical education instructors, speech or language therapists, music or art specialists, and others. Modern educators are largely not trained to interact with learners as whole and integrated beings, and even less are they trained to consider that the learner’s various domains may have a common upstream source of regulation in the brain.

The allostatic educator is dynamically flexible and uses different educational approaches in ways that fit the need. During the earliest phases of life, there is major biological development in neural systems that may be well supported by organic educational practices. Education for cognitive acquisition or cultural transmission supports learners to “stand on the shoulders of giants” with respect to established questions. In its essence, allostasis is especially aligned with constructivist educational practices – when learners are ready to question the question itself – which are attuned to the contingent and changing nature of skills and knowledge. By the lights of allostatic neuro-education, the most well educated student is not the one with the highest scores on fixed and standardized tests, but the one who is most robustly positioned to create successful interactions with complex, variable, and unpredictable environments. Capacity for such success has evolutionary consequences for both the individual and society.

At this juncture we consider whether the proposition to use allostasis as the basis for a new vision of education represents a form of the naturalistic fallacy. That is to say, though change may be the norm of nature, and though the brain’s biological role may be to serve as the organ of central command, it may not necessarily be warranted to presume that educators should use these concepts as normative guidelines for educational practice. For example, biological evolutionary theory has been used notoriously, and fallaciously, to justify policies that are intrinsically political (and often repressive), including social darwinism and eugenics. To this question, the authors answer (with further discussion in the following section) that the GANE has an explicitly philosophical dimension, and it specifically aims for attainment of a state of advanced executive functionality associated with authentic *freedom*. Freedom is represented by maximal *optionality* for both the learner and educator. Allostatic neuro-education is based on the power to *opt*, not the imperative of the *ought*. Optionality includes the freedom to place a relative emphasis on cognitive acquisitional educational practices that are themselves essentially homeostatic. It is rather the homeostatic paradigm that tends to commit the naturalistic fallacy, in conceiving that the constant (or typically average) set points for biological regulation should be considered normative, and therefore defended by means of therapeutic or otherwise normalizing interventions.

## Perspective, Objective, and Strategy of the GANE

We define allostatic neuro-education as *activity intended to educe (lead out) the full potentiality of learners, with respect for the integrated developmental trajectory of their multi-faceted brains, to support their successful engagement with complex, changing, and unpredictable environments.* Allostatic neuro-education may derive, now or in the future, from a range of principles and practices in education, child development, neuroscience, brain-focused technologies, or other fields. In this section we give initial substance to the GANE under the rubric of a perspective, an objective, and a strategy.

### The GANE Perspective is the Learner’s Extended Neurodevelopmental Trajectory

In bringing a developmental perspective to the foreground, the GANE aligns with pedagogical strategies introduced by a series of educators and psychologists beginning in the late 19th century. Rudolf Steiner (1861–1925) founded the Waldorf School in 1919, based on an appreciation of multiple domains of the human being that develop over the course of 7-years periods. Waldorf teachers progress with the same students from year to year so they can carefully steward the child’s long-term process for completing these tasks, and educational practices are designed to encompass organic, cognitive acquisitional, and constructivist objectives. John Dewey (1859–1952) proposed that education should be experiential and guide learners to develop constructive engagement with their surroundings including their larger societal context. Jean Piaget (1896–1980) modeled human cognitive development as proceeding through stages, from basic sensory impressions and motor activities, to a pre-operational stage that includes language acquisition, to concrete operations which include a capacity for logic, and finally to a formal-operational stage marked by abstract reasoning. Lawrence Kohlberg (1927–1987) showed that learners developed moral reasoning in a sequence of stages from self-interest (pre-conventional morality), to social consensus (conventional morality), to universal principles (post-conventional morality). Erik Erikson (1902–1994) conceptualized the life cycle to include a series of thematic tasks whose successive completion was required for subsequent stages of development.

Broadly, considerations for the educator as steward of development are threefold. Probably the most important consideration, the one that has historically been appreciated as maternal wisdom but which is now receiving extensive empirical validation, is that early life exposure to adversity or toxic stress levels confers a major risk for negative outcomes in childhood and beyond, due to disruptive impacts on systems for learning, health, and behavior (Shonkoff et al., 2012). Deleterious effects of repeated biochemical stress reactions have been referred to as allostatic load (McEwen, 1998; Juster et al., 2010), and the effects of childhood adversity on adult outcomes are independent of established adult-status risk factors (Danese and McEwen, 2012). Secondly and from a pedagogical perspective, the educator as steward of development should be sensitive to exposing learners to challenges prematurely. For example, attempts to impart abstract concepts too soon may yield poor grasp of those concepts, while robbing the learner of opportunities for experiences salient to their stage. Thirdly, incomplete maturation or failure to complete a task at a given stage (often in association with adversity or toxic stress) may hinder subsequent progress, just as a weak foundation is problematic for constructing higher floors of a building.

On the basis of scientific consensus and in alignment with the paradigm of allostasis, the importance of a developmental perspective is supported by the Research Domain Criteria (RDoC) initiative of the US National Institutes of Mental Health (NIMH). Decades of research have elucidated relationships between brain functionality and behavior, and the NIMH introduced the RDoC in 2009 as a way to advance understanding of mental and behavioral health by transitioning from symptom and behavior-based diagnostic systems to a framework that focuses on underlying brain biology (Cuthbert and Insel, 2013). The initial core domains of brain functionality identified by RDoC are arousal (including sleep), positive valence (including appetitive or reward-oriented behaviors), negative valence (including fear or anxiety), cognitive systems (for attention, memory, language), and systems for social processes (including affiliation and social communication). These core domains are operative in both health and disease, and they cut across different diagnostic categories. RDoC is poised to support advance in mental and behavioral health by suffusing the approach of researchers and practitioners with conceptualizations and interventions – even those that are entirely psychosocial in character – that are based on appreciations of brain functionality. Critically, developmental trajectories exist for all the major brain domains defined by the RDoC (Casey et al., 2014). These trajectories are impacted by use and environmental influences, and they can be presumed to influence one another.

Like behavioral health research and care, the field of education may also be poised to benefit from conceptualizing the learning child as a unique being with a unique brain, with core domains in various stages of development. As alluded to in Homeostasis, Allostasis, and Education, the human brain undergoes massive changes from embryogenesis to the post-natal period, and it remains plastic throughout the lifespan, presenting an array of conjecturable implications for educators as stewards of development. Neural systems for executive function and cognitive control do not fully mature until the third decade (Hsu et al., 2014), lending credence to organic educational views that use metaphors of blooming or pruning through late adolescence and beyond. Adolescence is characterized by a marked (quadratic) increase in incentive motivation (Luciana, 2013) that bears upon the developing reward system with implications that have been studied for mental health, but this trajectory might also be explicitly considered and leveraged by educators. Though the existence of a critical period for second language acquisition has been questioned (Vanhove, 2013), early education for bilingualism may confer a variety of benefits for cognitive development, including increased cognitive control and possibly generation of cognitive reserve (Bialystok et al., 2012). Alteration of circadian rhythm or sleep, an aspect of arousal, is common in adolescence and may disrupt executive functionality and reward processing (Hasler et al., 2012; Telzer et al., 2013), and advancing strategy and policy to support optimal sleep across the life span is a sensible priority for any approach to neuro-education (Sigman et al., 2014).

To further illustrate the contrasting approaches of cognitive acquisition-focused education and the neurodevelopmental perspective of the GANE, we consider the hypothetical case of a child who is found to be consistently inattentive, distractible, and fidgety. In a homeostatic, cognitive acquisition learning context, this child may be recommended or referred, by an educator, a physician, or parents themselves, to undergo consultation with a behavioral health specialist. If the child falls outside consensus criteria for normal attention and behavior including executive functionality, he or she may be given a diagnosis of attention deficit-hyperactivity disorder (ADHD). Non-normality may be attributed to a variety of disturbances in underlying neural mechanisms that, though possibly understood to have phenotypic expression along a continuum, nonetheless result in categorical assignment to a status of disease versus non-disease. This evaluation has taken place in a *spatial* dimension, wherein fundamentally the individual is compared, at a given moment in time, to other individuals.

The GANE instead evaluates the child’s behaviors or tendencies primarily as a snapshot within a longitudinal frame of complex neurodevelopmental trajectories. That is to say, the GANE is critically sensitive to the *temporal* dimension in its evaluation, such that the individual is appreciated primarily with respect to his or her own past and potential futures. Differences in the stepwise unfolding of brain domain functionalities may have consequences across trajectories. The pioneering work of Harry Harlow, for example, showed the importance of healthy attachment for later life development as a whole, and a recent report (Roskam et al., 2014) has shown an association between duration of exposure to early attachment deprivation (likely impacting brain domains for social affiliation, arousal, and affective valence) and degree of ADHD symptoms in adolescence. These data reinforce that brain domains interact with one another over time, and that ADHD cannot be conceptualized exclusively as a disorder of executive functioning. Furthermore while individuals with attention deficits may have altered sensitivity in the reward system that may confer a later risk for addictive disorders (Blum et al., 2008), it is possible that this same difference may, for some, contribute to success in risk-related endeavors that can have higher-order societal impact, including entrepreneurship. The GANE developmental view requires a willingness to use the biological imagination, for educators to create mental pictures related to the learner’s neural past and future. Such an approach promotes greater respect for the learner’s full life, both backward and forward in time, and appreciation of both risks and opportunities.

Implications of a neurodevelopmental perspective are even more significant with respect to interventional strategies. The spatial (cross-sectional) context of homeostatic understanding invokes the need for interventions that can correct putatively dysfunctional neural mechanisms, bringing them in alignment with the average or putative norm. In contrast the temporal (longitudinal) context of the GANE encourages intervention for self-calibrated advance that respects the unique needs of an individual’s own given state. In the first place, a nuanced view of the temporal dimension should presume the existence of natural variability for development across all domains of brain functioning, no different from variability in ages for children to undergo physical growth spurts. Such a view should lend a degree of conservatism against interventionist strategies that may have non-trivial risk profiles. More to the point, the GANE educator is at liberty to experiment with ways to support or remediate the learner’s attention and behavior – through adjusting educational activities, demand levels, situational contexts, or other strategies – to discover approaches that are effective for the learner’s unique needs. Developmental principles may even require regressive movement (“one step back, two steps forward”) that enables re-engagement with earlier tasks or stages that were uncompleted or otherwise disturbed in their unfolding. Such constructive trial and error or complex remediation may be difficult in many state-administered mass educational settings – where requirement for a statically high attention level and expectations for learning are highly standardized and normed – but they are realistic in organic educational traditions, for example homeschooling, the one-room schoolhouse, or other educational settings in which teachers progress with learners from year to year.

### The GANE Objective is to Enable the Experience of Authentic Freedom

What makes Homo sapiens most distinctive and unique in the animal kingdom is our prefrontal cortex, the brain region that supports advanced cognitive skills and executive functionality (Goldberg, 2001). The prefrontal cortex mediates capacities for imagination, reasoning, inhibition of impulses, and evaluation of the salience of competing stimuli (both internal and external). The capacity for conscious choice and decision-making permits a qualitatively and quantitatively advanced degree of power over other biological sub-systems, behavior, and the environment at large. As a species we have substantially removed many of the natural pressures which historically influenced our likelihood for survival. Our species status now depends more on our creative interaction with each other and the products of our own prior creation, and our degree of freedom is such that we are now able to influence our own evolution. The prefrontal cortex, mediating the capacity to bootstrap, to re-appraise situations in continuously novel and adaptive ways, to respond rather than react, and to do all the above in the service (if we choose) of higher-order goals, is undoubtedly a critical neural substrate for allostasis itself.

Despite this potentiality, education as homeostatic cognitive acquisition tends to focus on a relatively circumscribed understanding of healthy executive functionality. Modern educational systems typically intend for learners to acquire relatively standardized cognitive skills and knowledge. The GANE instead conceives that executive functionality of a caliber reflective of advanced human creativity and flourishing – as expressed in the ideals of the constructivist – entails a capacity for clarity of perception and thought, discernment, use of the imagination, and exercise of self-regulation and willful action that collectively are likely to require a symphony of functioning across brain domains.

In this context, we highlight the contribution of Rudolf Steiner as a philosopher (which is the basis of his contribution as a constructivist educator). Steiner maintained that a capacity for *intuitive thinking*, in which *concepts* are united with *percepts* (which include feelings), is the prerequisite for *moral imagination*, which is in turn the basis for *ethical individualism* (Steiner, 1894), which he considered to be the highest form of human attainment. For Steiner, the significance of ethical individualism lay in its expression of the human as a truly free being. Education for cognitive acquisition, being society-centric, is least obviously intended as a direct support for an individual’s authentic freedom. However, neither can learners limited to organic (or romantic) educational practice be considered truly free, in that they may be bound to a variety of emotions or physical impulses. Even many constructivist thinkers do not imply or adumbrate the advanced conception of freedom articulated by Steiner, in that they may restrict understanding of freedom to political dimensions, or place excess emphasis on social interactional processes or universalistic principles (including Kant’s categorical imperative) that may not be consistent with unique and changing exigencies of an individual’s particular life and natural context. With its focus on the human power for intuitive thinking – or cognition of concepts in concert with subtle somatosensory perception – Steiner’s philosophy of freedom exemplifies expression of competent, integrated, and expansive executive functionality whose emergence is the objective of the GANE.

The form of executive functionality envisioned by the GANE is thus of a variety more nuanced or complex than is commonly presumed adequate or even desirable by cognitive acquisitional education or homeostatic neuroscience, which are substantially aligned with positivist philosophical traditions that discount or reject introspection or intuition as a reliable source for understanding. Cognitive acquisitional education and homeostatic neuroscience tend to view the emotions or intuitions as the province of systematic bias, leading to cognitive error. Or these forms of subjectivity become a matter for consideration only after they have become grossly dysregulated, at which point pathways of clinical mental health evaluation and treatment may be deemed needful. In contrast, the GANE aims to integrate the emotions and interoceptions especially in their subtlety, and to leverage their positive and constructive role for the brain and the learner as a whole. In this regard, meaningful insights appear to be accumulating in the field of affective neuroscience. For example, Antonio Damasio (1994) proposed a “somatic marker” hypothesis which implies that emotional or somatosensory signals are needful for effective abstract reasoning. Interoceptive sensations of one’s body state (“gut feelings”) may serve as barometer for one’s arousal status, representing signals that have implications for safety or action, and that may be a core element of consciousness itself (Craig, 2009). Furthermore, emotional expression represents critical currency for interpersonal interactions, especially insofar as human relationships are often defined by qualitatively differentiated and calibrated degrees of autonomic arousal (Porges, 2011), and this topic is discussed further in the following section. Sterling (2014) has recently proposed that mental health should itself be defined as “responsiveness of the conscious and unconscious mind to the full range of signals from many sources.”

The objective of the GANE is thus to facilitate the emergence of an advanced form of executive functionality that recognizes, models and supports healthy forms of emotional or intuitive self-awareness, self-regulation and social relationship, and imagination. Less than full optionality to benefit from all these signals, without being overwhelmed by them, represents a deficit of human freedom. Organic as well as constructivist forms of educational practice, with their emphasis on well-being and pro-active interaction, may be well positioned to serve the GANE objective. Cognitive acquisitional educational practices too can support the GANE objective, especially if they include pedagogical strategies that aim for learners to have a *feel* for their acquired skills and knowledge. Normative inclusion of a healthy feeling dimension to educational practice represents a qualitative advance that should have non-trivial consequences for learners’ lifelong capacity to interact successfully with their environments. At a minimum, the GANE stands against stressful educational environments that generate an excessively competitive culture or are permissive to social bullying. Such settings will be unlikely to support capacities for subtle somatosensory perception and finely calibrated emotional awareness and expression, and in some cases they may have an outright poisonous influence on learning. This idea is further developed in the following section.

### The GANE Strategy is to Proceed with Sensitivity to Rhythms

The insights of the paradigm of allostasis can be restated to the following effect. The brain is the organ that oversees management of the variability of rhythms across systems, and it manages rhythms to increase the overall likelihood of successful interaction with complex environments. Dynamic adaptation or recalibration of system set points for optimal activity patterns is essentially indistinguishable from being in the right rhythm, at the right time, for the right purpose. Rhythmic dynamics exist across different domains of brain functionality, and at different scales of activity within those domains including gene expression, neuronal activation and synaptic neurotransmission, synchronous fluctuation of neuronal assemblies, bidirectional brain–body communication, and subjective experiences and observable behaviors. In particular, we propose that attunement to rhythms of *arousal* is likely to hold key value for the educator, and for purposes of the GANE we discuss arousal as the pattern of activity in the autonomic nervous system.

Historically, the sympathetic (“fight or flight”) and parasympathetic (“rest and digest”) divisions of the autonomic nervous system have been considered to act in paired antagonism. That is to say, these divisions have been conceived as functioning in a manner wherein an individual is either in a state of low or high arousal (parasympathetic or sympathetic activation, respectively). Of the two divisions, the sympathetic has been most commonly associated with states of disturbance, especially with respect to mental health (Roth et al., 2008), pain (Martinez-Martinez et al., 2014), and cardiovascular disease (Seravalle et al., 2014), and increased sympathetic activity has been demonstrated in young women with school burnout (May et al., 2015).

Alternatively, the polyvagal theory models autonomic functionality in a way that advances beyond the notion of paired antagonism between the sympathetic and parasympathetic divisions. To begin, polyvagal theory recognizes the existence of two anatomically and functionally distinct sub-systems of the parasympathetic division that derive from different phases of vertebrate evolutionary phylogeny (Porges, 2011). A myelinated branch of the vagus nerve (the main nerve of the parasympathetic division) is an evolutionarily advanced component of the autonomic nervous system, especially operative in humans, and it functions as a fine-tuned brake on high-arousal (fight/flight) mechanisms of the sympathetic division. The myelinated vagus permits self-calming and nuanced forms and degrees of emotional communication (and thus likely supports the advanced executive functionality that is the objective of the GANE). In contrast, an unmyelinated branch of the vagus, especially operative in early (reptilian) vertebrates, produces a “freeze state” for situations of novelty but also overwhelming stress. The unmyelinated vagus may be associated with neurogenic bradycardia (brain-directed slowing or even stopping of the heart), emotional numbing, or behavioral shutdown.

Polyvagal theory proposes that the functionality of these divisions is organized in a hierarchical way with a basis in evolutionary phylogeny. Normatively and in a non-threatening environment, the myelinated vagus should prevent wasteful high arousal mediated by the sympathetic division. If an environment is perceived to be dangerous, then myelinated vagal activity may give way to sympathetic fight/flight functionality to permit mobilization behaviors, so that a stressor can be physically overcome or escaped. If sympathetic fight/flight is inadequate, then unmyelinated vagal activity produces a freeze or shutdown mode of last resort. Humans will tend to “neurocept” different degrees of safety in different social and environmental contexts, and they may tend to calibrate their autonomic arousal toward one of these modes of functionality, without conscious intent. For safe, threatening (but tractable), or overwhelmingly stressful environments, autonomic regulation will tend to calibrate, respectively, toward parasympathetic fine-tuning, fight/flight arousal, or a parasympathetic freeze mode. All three modes or levels of arousal recognized by polyvagal theory are conserved in humans, and the different modes have pervasive influence on numerous aspects of behavior, performance, and health that are salient to the GANE.

In the first place and as a matter of primary pedagogical strategy, allostatic neuro-education aims for experientially palpable and subjectively enjoyable oscillation between parasympathetic fine-tuning and subtle degrees of sympathetic arousal. We propose that a rhythm of educational practice that is sensitive to alternation in these autonomic states has greater potential to educe the learner’s engagement, and in self-directed ways. Periods of excitement may ebb and flow with periods of calm reflection or incubation, in association with interoceptive functionality of the vagus nerve as described by polyvagal theory, to support awareness of one’s visceral sensations. Moreover, polyvagal theory recognizes that the brainstem nucleus of the myelinated vagus overlaps with cranial nerves that manage muscles for facial and vocal emotional expression, and auditory sensation. Descending activation of the myelinated vagus may support enhanced capacity for highly nuanced emotional communication with others. Thus parasympathetic fine-tuning, associated with safe environments, enables calm and subtle bidirectional brain–body communication and should facilitate appraisal of information in undelimited ways, to increase the learner’s cognitive engagement and optionality of response. It seems likely that a range of influences on executive functionality including physical movement (Khalil et al., 2013; Rommel et al., 2013), musical training (Kraus et al., 2014; Miendlarzewska and Trost, 2014; Fonseca-Mora et al., 2015; Francois et al., 2015), complex games (Kim et al., 2014), and exposure to natural environments (Taylor and Kuo, 2009) entail rhythmic or playful engagement with the learner’s autonomic arousal level, and these studies further highlight how brain functional domains interact in complex ways to produce lived and meaningful experience.

Secondly, polyvagal theory posits a need to recognize that not all low arousal is created equal. There is a major qualitative difference between the low arousal of an individual who has developed robust executive functionality for fine-tuned parasympathetic (myelinated vagal) self-calming in the face of stress, for example, and the evolutionarily ancient parasympathetic (unmyelinated vagal) freeze mode of someone who has not recovered from trauma. The former individual has leadership potential, whereas the latter is an individual who is likely to have low emotional self-awareness and may be impaired in their capacity for healthy social interaction. Calibration of autonomic activity for unmyelinated vagal freeze mode may be a pernicious if unrecognized reality in mass educational systems. This mode is likely to be problematic for learning, and it may be associated with emotional disengagement to the degree that it creates risk for maladaptive compensatory behaviors including anti-sociality (Lee et al., 2014).

The GANE strategy of attunement to rhythms of learning may have implications for reconsidering the burgeoning use of computing technologies in education. Computers have been widely promoted in education on the generally presumptive basis that the scope and speed of information afforded should translate into benefits for educational practice. In contrast, others voice caution about the use of technology in education, and this view is expressed in Steiner-inspired education. Steiner schools explicitly refrain from exposing learners to computing technology until the later middle grades or high school, on grounds that such influence may produce risk for detrimental effects on the learner as a whole. Instead the learning environment is focused on GANE themes introduced earlier, with attention to the child as a composite of multiple domains, using rhythm-sensitive exercises, creative arts and crafts, foreign language, music, natural environments, and physical movement, in ways that make maximal benefit of direct human engagement, in alignment with the implications of polyvagal theory, and with sensitivity to developmental context. Like Steiner schools, the GANE emphasizes the value of real-time human-to-human engagement as a way for the learner to use the teacher as a mirror or a guide to develop their capacity for autonomic fine-tuning and stress responsivity.

Nonetheless, neither the poly-vagal perspective nor the GANE imply that the computer *per se* is a negative influence in education. The key questions, as with all technology, instead pertain to whether and how computing technology can be best put to the most thoughtful and constructive use. For example, we hypothesize the likelihood of educational value from orchestration of real-time interactions between young learners in a rural environment and geographically distant teachers of a foreign language. Such computer-enabled learning interactions might add invaluable sophistication to education as cognitive acquisition (or cultural transmission), and also could support the GANE to achieve its highest constructivist objectives.

## Implications of the GANE

### Testable Hypotheses Related to the GANE

The GANE is not itself a theory in the sense of being a unitary and falsifiable conjecture. Rather it is a re-visioning of what education might represent, and how it might more faithfully serve both learners and society. The perspective that favors the learner’s long-term, whole-brain, whole-life trajectory, is a value choice. Likewise the objective of expansive executive functionality, aligned with Steiner’s contention that the highest human good that can be attained is ethical individualism, is a philosophical premise. Many learners, educators, and parents who gravitate toward the GANE may do so on the basis of their resonance with its principles, rather than because of empirical validation in controlled studies.

Nonetheless a variety of meaningful hypotheses relating to the core elements of the GANE can be generated and tested. Its strategy to attend to *rhythms*, and especially rhythms of arousal and experiential excitement, is perhaps the most amenable to relatively straightforward scientific exploration. The familiarity of this idea suggests it is a general-purpose strategy that could serve a variety of educational approaches as well as vocational needs. Numerous studies, including those mentioned in Section “The GANE Objective is to Enable the Experience of Authentic Freedom,” suggest that thoughtful inclusion of activities involving rhythmic behavioral or sensory attunement – for example physical education and athletics, music, drama, dance, and related pursuits – as elements of core curriculum, may enhance rather than detract from the achievement of educational objectives including those related to cognitive acquisition. Moreover, an expansive conception of “rhythm” invites hypotheses that explore highly technical understandings of *brain* rhythms and how they may relate to learning, and this topic is explored in Section “Case Studies Illustrating Use of Allostatic Neurotechnology.”

A range of hypotheses may be inspired by the long-term developmental perspective of the GANE. Studies may focus on developmental causes of a learning challenge, rather than symptomatic remediation. In particular, the role of toxic stress described in Section “The GANE Perspective is the Learner’s Extended Neurodevelopmental Trajectory” would appear to be a critical area for further investigation. It has been proposed that early childhood adversity and toxic stress should be approached with the seriousness that is accorded to major medical risk factors, and that pediatric medicine should take a leadership role on this front (Shonkoff et al., 2012). Sensitivity to the influence of stress on development should, however, also be an obvious priority in educational settings, where children spend far more time. Educators are well-positioned to lead or collaborate on hypothesis-driven studies intended to prevent or mitigate the consequences of toxic stress for the brain, body, learning, and behavior.

It is also possible to design studies that aim to identify the respective consequences of educational approaches that place varying degrees of emphasis on long-term developmental trajectories. For example in an evaluation of children enrolled in schools with different reading instruction ages (age 7 in a Steiner-based school, versus age 5 in a state-curriculum school), it was reported that by age 11 there were no reading fluency disadvantages for the children who began reading instruction at the later age (Suggate et al., 2013). Hypothesis-driven studies may also evaluate whether the presence of certain characteristics that are considered non-normative at a given time, may be associated with other forms of positive outcome at a later time in the learner’s trajectory.

The GANE objective of enabling the experience of authentic freedom is fundamentally philosophical, nonetheless it also entails testable hypotheses. For example, it has been hypothesized that learners educated on the basis of Steiner principles (or congruous educational systems) would have more self-efficacy than those learning on the basis of conventional (cognitive acquisitional) curricula, when transitioning to higher education (Shankland et al., 2009). The GANE objective also aligns with increasingly sophisticated hypotheses and experimental paradigms at the intersection of cognitive, affective, and social neuroscience, that aim for more detailed understanding of the multi-directional influences among bodily interoceptions, social interactions or cultural context, and cognitive processing and appraisal (e.g., Immordino-Yang et al., 2014). GANE educators who have a robust qualitative appreciation for learners in their multi-dimensionality (physical, emotional, mental, spiritual aspects) may be well positioned to dialog with neuroscientists to help formulate testable hypotheses that are thoughtfully sensitive to these domains. They may also be positioned to help develop study designs intended to identify brain states that educators might then use to help guide their pedagogy (Gabrieli et al., 2015).

### Case Studies Illustrating Use of Allostatic Neurotechnology

We contend that technological innovation can ease the path of allostatic neuro-education if the technology is itself guided by allostatic principles. Allostatic technology should aim to support individuals to be optimally adapted to their environmental context, in part by supporting system set points to be dynamically flexible and not fixed. Given the complexity of the brain, its dynamics, and its role for central command, allostatic technology aims to respect the brain’s own capacity for self-optimization, which may be expected to involve increased efficiency for learning. More technically, it has been proposed that allostatic interventions should restore responsiveness of neural systems to the full range of signals from many sources, and to do so by using “natural mechanisms for predictive regulation [that] involve continual updating of knowledge stores” (Sterling, 2014). By contrast, brain-centric technologies exist including electroencephalographic biofeedback (“neurofeedback”), transcranial magnetic stimulation, transcranial direct current stimulation, and others that are largely homeostatic in their reliance on normative (and externally given) standards for neural functioning, and/or strategy based on direct induction of corrections or changes to brain mechanisms. In this section we present two case studies that illustrate use of a non-invasive allostatic neurotechnology in ways that are aligned with the GANE and may support the formulation of controlled, hypothesis-driven studies that relate to the GANE and supportive technologies.

#### Case 1

An 18 years-old male, carrying diagnoses of Asperger’s syndrome and ADHD, was enrolled in an IRB-approved, open label exploratory study at Wake Forest School of Medicine, evaluating the use of an allostatic neurotechnology for a range of different purposes. The technology operates through rapidly updating the brain about its own oscillatory activity through the medium of sound and is intended to support brain activity to be flexibly adaptable around an individual’s unique oscillatory set points (Gerdes et al., 2013). The participant’s mother reported that he was always, “on edge, hyper-focused, and a light sleeper.” He attended several local private schools for elementary and middle school years, including one focused on students with learning challenges. He had been home-schooled for the last several years, and had just completed the school year at the time of enrollment. He had been using the stimulant lisdexamfetamine dimesylate (Vyvanse^®^), 30 mg daily, for management of attention deficit symptoms, for 2 years prior to enrollment. Under his physician’s guidance the stimulant was tapered to 1–2 times per week, and was then discontinued just prior to study entry. Supplements included astaxanthin, omega 3, blue-green algae, calcium, magnesium, and Co-Q 10.

After informed consent, the participant completed self-report symptom inventories including the Insomnia Severity Index (ISI), Beck Anxiety Index (BDI), Beck Depression Index – II (BDI-II), and the Autism Spectrum Quotient (AQ). An assessment of brain electrical frequencies and amplitudes was obtained, as previously described (Gerdes et al., 2013), consisting of 3 min recordings obtained from standard locations on the scalp (based on the 10–20 system), and including 1 min of recording for each of three eye states (eyes closed, partially closed, and eyes open). With eyes closed, the participant was at rest, while a cognitive task was performed during eyes open recording. The assessment provided a “snapshot” of relative symmetry between homologous brain regions, along with the distribution of amplitudes among different frequency bands at each location.

**FIGURE 2:**
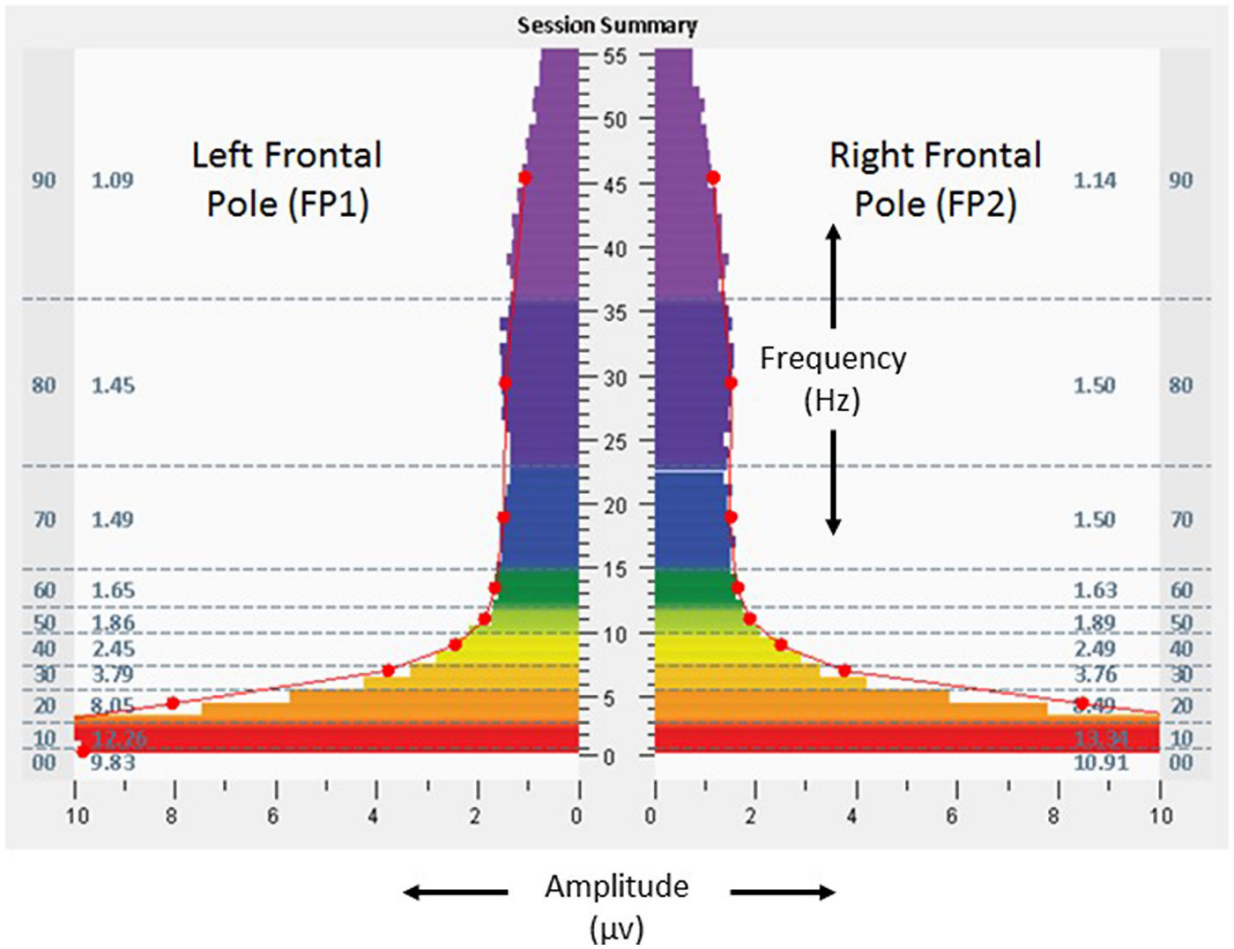
**Fast Fourier Transform spectral display of 1 min recording of brain electrical activity at left and right frontal poles (FP1 left, FP2 right), with eyes open, during the baseline assessment, from the 18-years-old learner described in the text.** Individual color bars reflect amplitude averages for 1 min of recording, eyes closed, at rest, without stimulation. Columns to the left and right of the color bars denote 10 frequency ranges of aggregated data (00: < 1.0 Hz; 10: 1.0–3.0 Hz; 20: 3.0–5.5 Hz; 30: 5.5–7.5 Hz; 40: 7.5–10.0 Hz; 50: 10.0–12.0 Hz; 60: 12.0–15.0 Hz; 70: 15.0–23.0 Hz; 80: 23.0–36.0 Hz; 90: 36.0–48.0 Hz) and numerical values for amplitude averages in those ranges.

**FIGURE 3:**
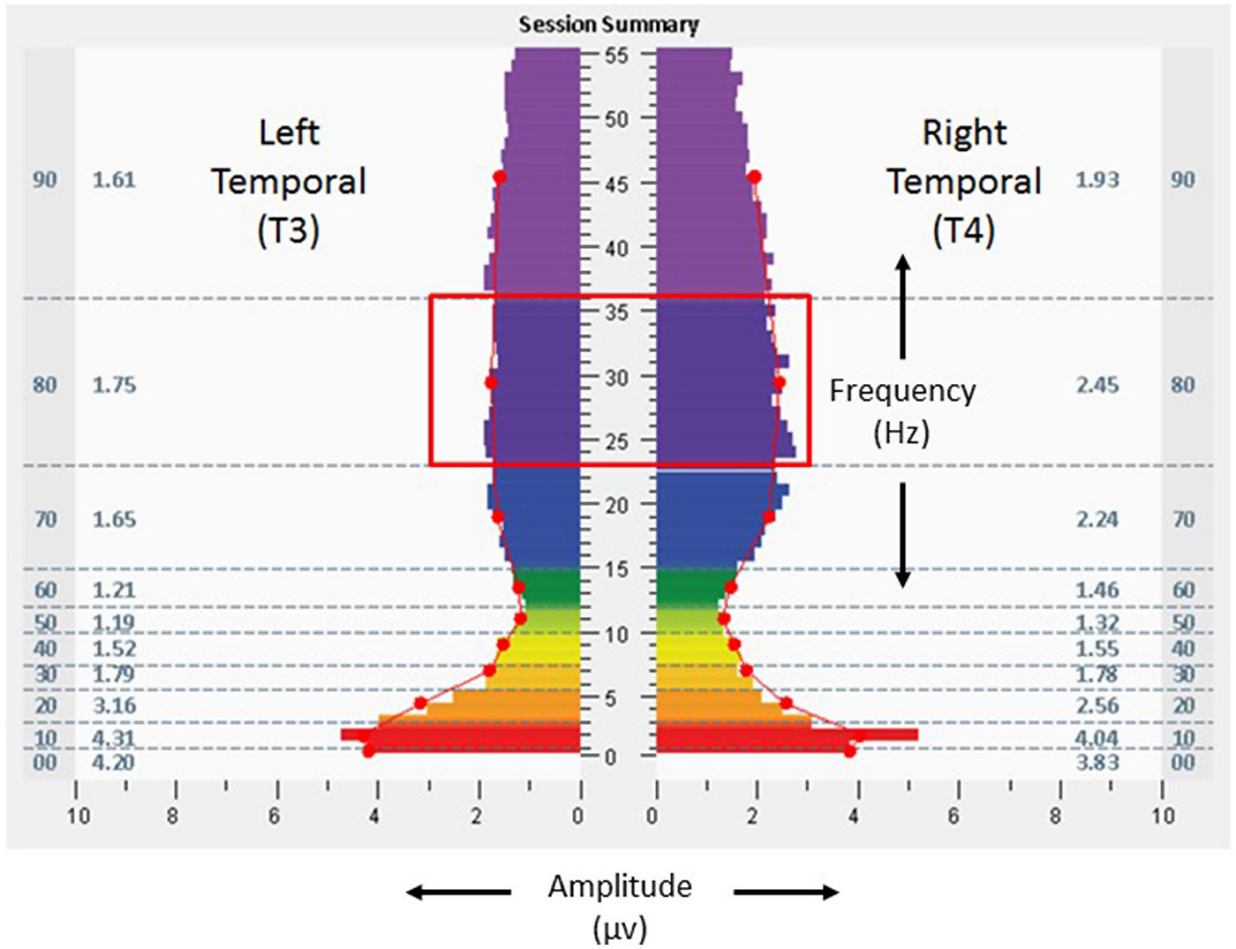
**Fast Fourier Transform spectral display of 1 min recording of brain electrical activity at the left and right temporal lobe (T3 left, T4 right), with eyes closed, during the baseline assessment, from the 18-years-old learner described in text.** Red box denotes the 23–36 Hz frequency range mentioned in the text. See **Figure 2** legend for detailed explanation of data elements.

Scores on baseline symptom inventories – ISI 6, BAI 14, BDI-II 11, and AQ 39 – suggested absence of clinically relevant insomnia, presence of mild anxiety, presence of depressive symptoms on the upper end of the normative range, and a strong likelihood of Autism Spectrum Disorder. Result for the BDI-II also included an elevated score for the measure assessing Concentration Difficulty (“It’s very hard to keep my mind on anything for very long”). **Figure 2** shows baseline assessment spectrographs of brain electrical activity based on 1 min recording at the frontal pole locations (FP1, left; FP2, right) with eyes open. The very low frequency ranges (0–1 Hz) were notable for amplitudes of 9.8 and 10.3 μv at FP1 and FP2, respectively. At the temporal locations (T3 left, T4 right) with eyes closed (**Figure 3**), baseline assessment suggested 39% T4 dominance in the high frequencies (23–36 Hz), with average amplitudes of 1.7 and 2.4 μv on the left and right, respectively, in the same frequencies.

**FIGURE 4:**
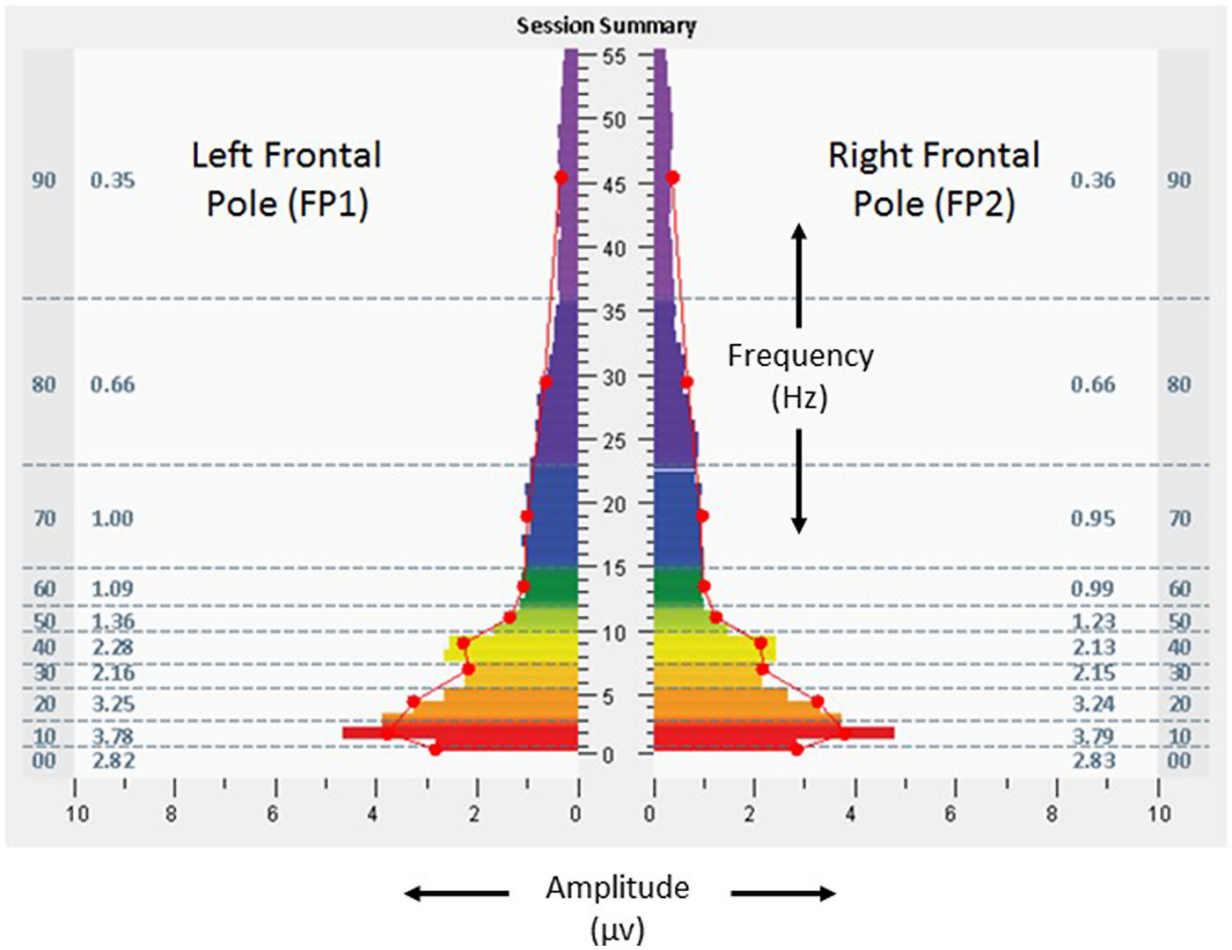
**Fast Fourier Transform spectral display of brain electrical activity observed during the penultimate minute of the penultimate allostatic technology session at the left and right frontal poles (FP1 left, FP2 right), with eyes open, from the 18 years-old learner described in the text.** See **Figure 2** legend for detailed explanation of data elements.

**FIGURE 5:**
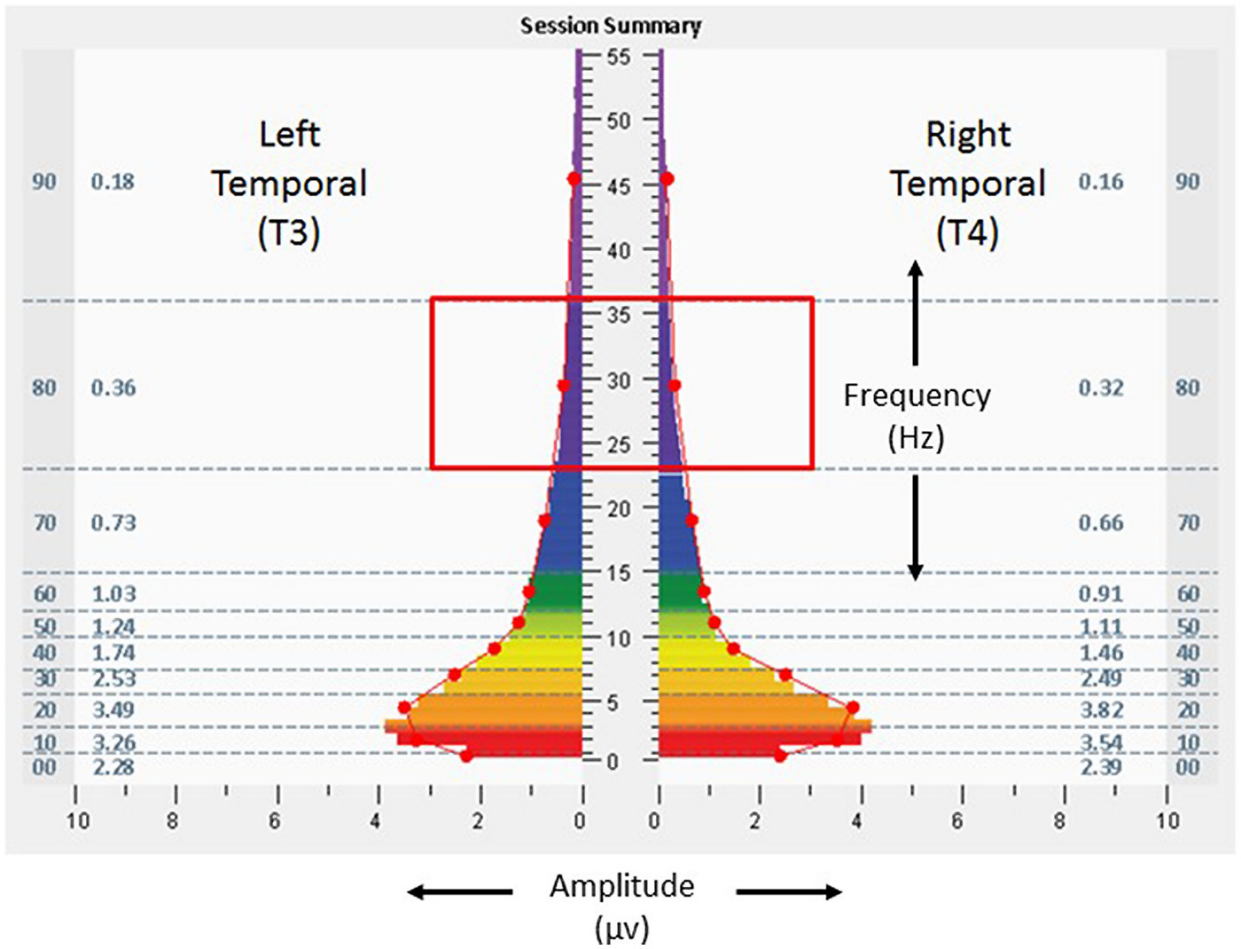
**Fast Fourier Transform spectral display of brain electrical activity observed during the penultimate minute of the penultimate allostatic technology session at the left and right temporal lobe (T3 left, T4 right), with eyes closed, from the 18 years-old learner described in the text.** Red box denotes the 23–36 Hz frequency range mentioned in the text. See **Figure 2** legend for detailed explanation of data elements.

The participant undertook 14 sessions with the allostatic neurotechnology (13 days of sessions, median of 76 min, range 64–84, with total protocol time of 1,066 min) over a total period of 40 days, with a 27 days recess between sessions 10 and 11. Sessions were well tolerated, with no adverse events reported. **Figures 4** and **5** provide spectrographs of data from the penultimate minute, of the penultimate session, providing insight into shifts that occurred during the course of the intervention. Electrical amplitudes were reduced across the frequency spectrum at bilateral frontal poles, and in the very low frequency (0–1 Hz) range they were an average of 2.8 μv bilaterally. At bilateral temporal lobes there were reduced amplitudes in the 23–36 Hz range (0.36 μv on left, 0.32 μv on right), with 13.2% left dominance at the same frequencies, representing a reduction in asymmetry.

At the post-intervention follow-up data collection visit (12 days after completion of sessions), the participant offered that he “felt better” and that “something seemed different.” His mother reported, “His teachers for summer programs noted his concentration and focus were better in class, as well as his willingness to participate.” The mother also noted that he was sleeping better and more soundly. He remained off the stimulant medication. Follow-up scores on symptom inventories – ISI 1, BAI 13, BDI-II 1, and AQ 40 – reflected improvement in sleep and reduction of depressive symptoms. For the BDI-II measure on Concentration Difficulty, the participant no longer endorsed concentration as a problem. Additional telephone follow-up with the mother, 4.5 months after completing the intervention, revealed that the participant was now enrolled in a special education program at a local high school, which included mainstream classes with the general school population. He had joined the local chapter of the ROTC and was being praised for his performance. She reported that the participant had remained off of the stimulant for 3 months, but that he had been re-started at 15 mg per day, half the prior dose, 5 days per week, as school started.

As discussed in Section “Perspective, Objective, and Strategy of the GANE,” homeostatic neuropsychiatry views attention-deficit hyperactivity disorder as a categorical diagnosis reflecting aberrant neural mechanisms. Alternatively, the GANE views cognition and behavior as being expressions that pertain to an individual within the context of their unique neurodevelopmental trajectory. The adolescent in this case was supported with approaches that reflect both strategies – educational settings aimed for sensitivity to the role of environmental factors on learning, and also use of stimulant medication aimed to increase (clamp) attention toward a more normative set point. In this case study, adjunctive use of allostatic neurotechnology was associated with self-adjustments in cognitive systems (attention), arousal (sleep), and social affiliation (class participation). High amplitudes in a very low frequency range (0.02–0.2 Hz) of neural oscillations have been described as an endophenotype of ADHD that relate to the default mode network (Broyd et al., 2011), and stimulant use in adults with ADHD has been associated with reduction in inattention and attenuation of very low frequency amplitudes (Cooper et al., 2014). Neural oscillatory changes represented as reductions in low frequency amplitudes at bilateral frontal poles in this individual may have been related to improved capacity for attention. Increased amplitudes in high frequency ranges have been described as a basis for insomnia (Perlis et al., 2001), raising the possibility that improved sleep quality in this subject was related to reductions of amplitude in the high frequency (23–36 Hz or higher) ranges demonstrated in both frontal pole and temporal regions. The participant’s baseline rightward dominance in temporal high frequency electrical asymmetry may have been suggestive of relative sympathetic tendency in autonomic regulation (Tegeler et al., 2015), and reduction of this asymmetry may have reflected calibration of his stress responsivity toward greater parasympathetic (myelinated vagal) self-regulation.

Stimulant medications have effects on neurodevelopmental trajectories for mood, stress responsivity, and reward processing (Marco et al., 2011; O’Daly et al., 2014). They are neurotoxic (Goncalves et al., 2014) and appear to impair neuroplasticity (Urban and Gao, 2014), and parents are shown to have apprehensions about their use for ADHD (Ahmed et al., 2013). In contrast to this cautionary view, others have proposed that homeostatic neuro-pharmacological intervention may be salutary for development, on the basis that targeted drug intervention may reprogram developmental trajectories so as to enable preventative cure of conditions such as ADHD and depression (Andersen and Navalta, 2011). To date, long-term follow-up of children treated for ADHD has not shown benefits for titrated psychoactive stimulant usage (or behavioral intervention, or their combination) over community-based care, on either academic, social, or clinical mental health outcomes (Molina et al., 2009).

#### Case 2

A 9 years-old girl in her fourth grade at a public school was noted to have “inadequate response to individualized educational interventions” and so was referred for evaluation by an educational diagnostician. She was assessed to have a specific learning disability related to reading and was placed in a Special Education Program. Her mother was advised that she should expect for her daughter to remain in this program for the duration of her formal schooling (through high school). The following summer she experienced a traumatic emotional loss when her maternal aunt died, at which time according to her mother she began experiencing emotional difficulties, including tendencies for anger and being “closed off.” Her medical and behavioral health history included enuresis (bed-wetting) and seasonal allergies. She used no medications. In the spring of her fifth grade, the child’s mother arranged for a series of 14 sessions using the same allostatic neurotechnology described in Case 1 (Gerdes et al., 2013), in the hope that doing so would support improvement in her reading comprehension, and also for possible emotional benefits. Initial effects noted by the mother included a greater degree of calm, being “not so wound up,” and more openness. Within about 9 months after the initial sessions, the mother felt that her learning capacity had increased markedly, with improvements demonstrable as faster speed for processing information. Behaviorally, the mother noted that the child had no episodes of enuresis for 9–10 months after starting sessions, followed by a recurrence of episodes that appeared to be related to anticipation of the end of school. The child underwent five more sessions in the winter of sixth grade. By the end of seventh grade, the mother’s impression was that the child was demonstrating significant improvements in reading comprehension. She was composing reports easily and in ways that included advanced word uses, and could communicate orally without stuttering. In the beginning of her eighth grade, the child’s skills were evaluated to be in a range that did not require the Special Education Program, and she began the year in a regular eighth grade classroom. Her initial marks (August 28, 2014) were English B+, Mathematics B, Physical Education A+, Science B+, and Bilingualism A+.

The child in Case 2 appeared to demonstrate a combination of behavioral and learning difficulties that may have been exacerbated by an emotional trauma, highlighting the influence of life events across brain domains, and possibly indicating that she was stalled at a developmental stage. Adults for example with complicated grief have been found to have deficits in cognitive function and structural brain changes (Saavedra Pérez et al., 2015). Nocturnal enuresis is reported to be associated with autonomic dysregulation (Yakinci et al., 1997). In this case study, use of allostatic neurotechnology was associated with improvement in emotional well-being as well as relief from enuresis, and these shifts are conceivably related to recovery from effects of emotional stress (Lee et al., 2014) that may have had implications for learning ability.

## Summary and Conclusion

Early and other approaches to neuro-education have used the metaphor of a bridge (Bruer, 1997; Sigman et al., 2014), which implies a fixed distance between the educator and the neuroscientist. In contrast, the present paper proposes that educational leaders and brain specialists who are like-minded in a developmentally sensitive and constructivist orientation, can collaborate now on groundwork that supports a new vision of learning. The GANE adduces the evolutionary model of physiological regulation known as allostasis, to flexibly apply a range of educational practices including the organic (home-schooling and other child-centric methods), cognitive acquisition (attainment of common understandings, measured through testing), and the constructivist (to enable dynamic forms of individual-societal interaction and renewal). Allostatic neuro-education recognizes change and not constancy to be the norm, defines success within the context of complex and changing natural environments as opposed to controlled settings, and identifies the brain as the organ of central command. In this paper we have characterized the GANE by introducing its perspective, its objective, and its strategy. The perspective is to focus on the learner’s full neurodevelopmental trajectory, rather than the immediate dictates of standardized testing. The objective is to support the emergence of competent, integrated, and expansive executive functionality, that supports the highest expression of humans as free beings. The strategy is to guide learning with attention to rhythms, especially rhythms of arousal, and to do so in ways that support palpable excitement. A variety of testable hypotheses derive from the GANE, and there may be particular need for studies of ways to prevent or mitigate the consequences of childhood adversity or toxic stress. Allostatic neurotechnology may support the GANE by respecting the brain’s complexity and supporting its capacity for self-optimization.

Life at any time is unpredictable, and much of its wonder is that the most unpredictable and unimaginable events are the ones most likely to produce the changes of genuine significance (Taleb, 2007). Recognition of this reality may lend greater weight to the constructivist aphorism that “the best way to predict the future is to create it.” Serious appreciation of unpredictability furthermore compels us to prepare new generations of learners – and their developing brains – to develop capacity to be adaptable in new ways and to greater degrees. In contrast, promotion of education as a way to support economic growth may be dubious strategy. Not only does such emphasis tend to demoralize both learners and educators, empirical study has found that, contrary to much public policy discourse, increasing education does not lead to increased economic productivity (Pritchett, 2001). In context of the accelerating change currently being experienced across societal sectors and around the globe, the aim of the GANE is to breathe new life into educational practice by educing the full potential of learners, through constructive appreciation of the complex and ever-changing human brain.

## Conflict of Interest Statement

The authors declare that the research was conducted in the absence of any commercial or financial relationships that could be construed as a potential conflict of interest.
